# High-quality draft genome sequence of *Rhizobium mesoamericanum* strain STM6155, a *Mimosa pudica* microsymbiont from New Caledonia

**DOI:** 10.1186/s40793-016-0212-4

**Published:** 2017-01-17

**Authors:** Agnieszka Klonowska, Aline López-López, Lionel Moulin, Julie Ardley, Margaret Gollagher, Dora Marinova, Rui Tian, Marcel Huntemann, T.B.K. Reddy, Neha Varghese, Tanja Woyke, Victor Markowitz, Natalia Ivanova, Rekha Seshadri, Mohamed N. Baeshen, Nabih A. Baeshen, Nikos Kyrpides, Wayne Reeve

**Affiliations:** 1 IRD, Cirad, Univ. Montpellier, Interactions Plantes Microorganismes Environnement (IPME), 34394 Montpellier, France; 2 IRD, UMR LSTM-Laboratoire des Symbioses Tropicales et Méditerranéennes, 34398 Montpellier cedex 5, France; 3 School of Veterinary and Life Sciences, Murdoch University, Murdoch, WA Australia; 4 Curtin University Sustainability Policy Institute, Curtin University, Bentley, WA Australia; 5 DOE Joint Genome Institute, Walnut Creek, CA USA; 6 Biological Data Management and Technology Center, Lawrence Berkeley National Laboratory, Berkeley, CA USA; 7 Department of Biology, Faculty of Science, University of Jeddah, Jeddah, Saudi Arabia; 8 Department of Biological Sciences, Faculty of Science, King Abdulaziz University, Jeddah, Saudi Arabia

**Keywords:** Root-nodule bacteria, Nitrogen fixation, *Rhizobium*, *Alphaproteobacteria*, *Mimosa*

## Abstract

*Rhizobium mesoamericanum* STM6155 (INSCD = ATYY01000000) is an aerobic, motile, Gram-negative, non-spore-forming rod that can exist as a soil saprophyte or as an effective nitrogen fixing microsymbiont of the legume *Mimosa pudica* L.. STM6155 was isolated in 2009 from a nodule of the trap host *M. pudica* grown in nickel-rich soil collected near Mont Dore, New Caledonia. *R. mesoamericanum* STM6155 was selected as part of the DOE Joint Genome Institute 2010 Genomic Encyclopedia for Bacteria and Archaea-Root Nodule Bacteria (GEBA-RNB) genome sequencing project. Here we describe the symbiotic properties of *R. mesoamericanum* STM6155, together with its genome sequence information and annotation. The 6,927,906 bp high-quality draft genome is arranged into 147 scaffolds of 152 contigs containing 6855 protein-coding genes and 71 RNA-only encoding genes. Strain STM6155 forms an ANI clique (ID 2435) with the sequenced *R. mesoamericanum* strain STM3625, and the nodulation genes are highly conserved in these strains and the type strain of *Rhizobium grahamii* CCGE501^T^. Within the STM6155 genome, we have identified a *chr* chromate efflux gene cluster of six genes arranged into two putative operons and we postulate that this cluster is important for the survival of STM6155 in ultramafic soils containing high concentrations of chromate.

**Electronic supplementary material:**

The online version of this article (doi:10.1186/s40793-016-0212-4) contains supplementary material, which is available to authorized users.

## Introduction

The ability of legumes to engage in a dinitrogen fixing symbiosis with soil dwelling bacteria, collectively known as rhizobia, has contributed to their success in colonizing nitrogen deficient soils over a broad range of edaphic conditions. While legume crops and pastures make important contributions to agricultural productivity, invasive legume weeds such as *Mimosa pudica* L. have a negative impact on natural and agricultural ecological systems. *M. pudica* originates from America [1] and became a highly invasive pantropical weed. It has been identified as a pest species, associated with land degradation, biodiversity loss, and reduced agricultural and therefore economic productivity, with attendant social and health impacts [2]. It requires resource-intensive chemical and mechanical control methods [2]. Conversely, however, it has potential commercial value as a source of silver nanoparticles and pharmacologically active phytochemicals, and as a phytoremediant for arsenic-polluted soils [3–6]. Understanding the *Mimosa* symbiosis can therefore help to achieve outcomes such as preventing biodiversity loss and improving the use of terrestrial ecosystems, as well as promoting sustainable industry, which form part of the Sustainable Development Goals adopted in September 2015 as part of the UN’s development agenda ‘Transforming our world: the 2030 Agenda for Sustainable Development’ [7].

*M. pudica* has the unusual property of interacting with microsymbionts belonging to both alpha- and beta-rhizobia [8, 9]. Alpha-rhizobia are preferred symbionts of most legume species, but beta-rhizobia have a far narrower host range, with a particular affinity for the *Mimosa* genus in South America [10] and endemic papilionoid species in South Africa [11]. Diversity studies have shown that alpha-rhizobia are found less frequently than beta-rhizobia in the nodules of *M. pudica* [12–17], and nodulating species exhibit different competitive and symbiotic characteristics [18, 19]. *M. pudica* thus represents an interesting legume species for comparative analyses of symbiotic traits and plant-infection genetic programs in the two categories of symbionts.

*M. pudica* was introduced to New Caledonia at the end of the 19^th^ century [15]. *Rhizobium mesoamericanum* STM6155 was isolated from nodules of *M. pudica* growing in soil characterized by neutral pH (6.8) and very high total nickel concentrations (10.1 g.kg^−1^) that was collected near the abandoned nickel mining site of Mont Dore (S3: 22°15’16.51”S and 166°36’44.27”E) in New Caledonia [15].

The 16S rRNA and *recA* house-keeping genes of STM6155 showed 100 and 97% nucleotide identity with their orthologs in *Rhizobium mesoamericanum* CCGE501^T^ from Mexico [20], and STM6155 was thus tentatively included in the same species. Among described alpha-rhizobial symbionts of *M. pudica* (*R. etli* bv. *mimosae*, *R. tropici* and *R. mesoamericanum*), *R. mesoamericanum* is the most frequently detected species, with a distribution on different continents (Central & South America, Asia) [17, 20]. In Mexico, endemic *Mimosa* spp. growing in weakly acidic, neutral or slightly alkaline soil are preferentially nodulated by Alphaproteobacterial rhizobia, including strains of *R. mesoamericanum* [21], whereas acid-tolerant *Burkholderia* spp. are favoured microsymbionts of endemic *Mimosa* spp., including *M. pudica*, in acidic Brazilian soils [14, 22]. *R. mesoamericanum* is much less effective for nitrogen fixation on *M. pudica* than *Burkholderia phymatum* STM815 or *Cupriavidus taiwanensis* STM6070 [12, 15], and much less competitive in comparison to *B. phymatum* and *B. tuberum* [19]. These data question how *R. mesoamericanum* can maintain itself as a symbiont of *M. pudica* despite its low competitiveness. Strain STM6155 has therefore been selected as part of the DOE Joint Genome Institute 2010 *Genomic Encyclopedia for* *Bacteria* *and Archaea-Root Nodule Bacteria* (GEBA-RNB) sequencing project [23, 24], to investigate the genome traits that enable this species to adapt to a symbiotic and saprophytic lifestyle. Here we present a summary classification and a set of general features for *R. mesoamericanum* STM6155, together with a description of its genome sequence and annotation.

## Organism information

### Classification and features

**Fig. 1:**
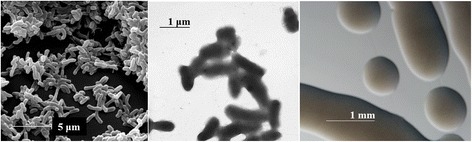
Images of *Rhizobium mesoamericanum* STM6155 using scanning (*Left*) and transmission (*Center*) electron microscopy and the appearance of colony morphology on solid media (*Right*)

*Rhizobium mesoamericanum* STM6155 is a motile, Gram-negative, non-spore forming strain in the order *Rhizobiales* of the class *Alphaproteobacteria*. The rod-shaped form has dimensions of 0.4–0.6 μm in width and 1.0–1.4 μm in length (Fig. 1 Left and Center). It is fast growing, forming colonies within 3–4 days when grown on half strength Lupin Agar (½LA) [25], tryptone-yeast extract agar (TY) [26] or a modified yeast-mannitol agar [27] at 28 °C. Colonies on ½LA are white-opaque, slightly domed and moderately mucoid with smooth margins (Fig. 1 Right).

**Fig. 2:**
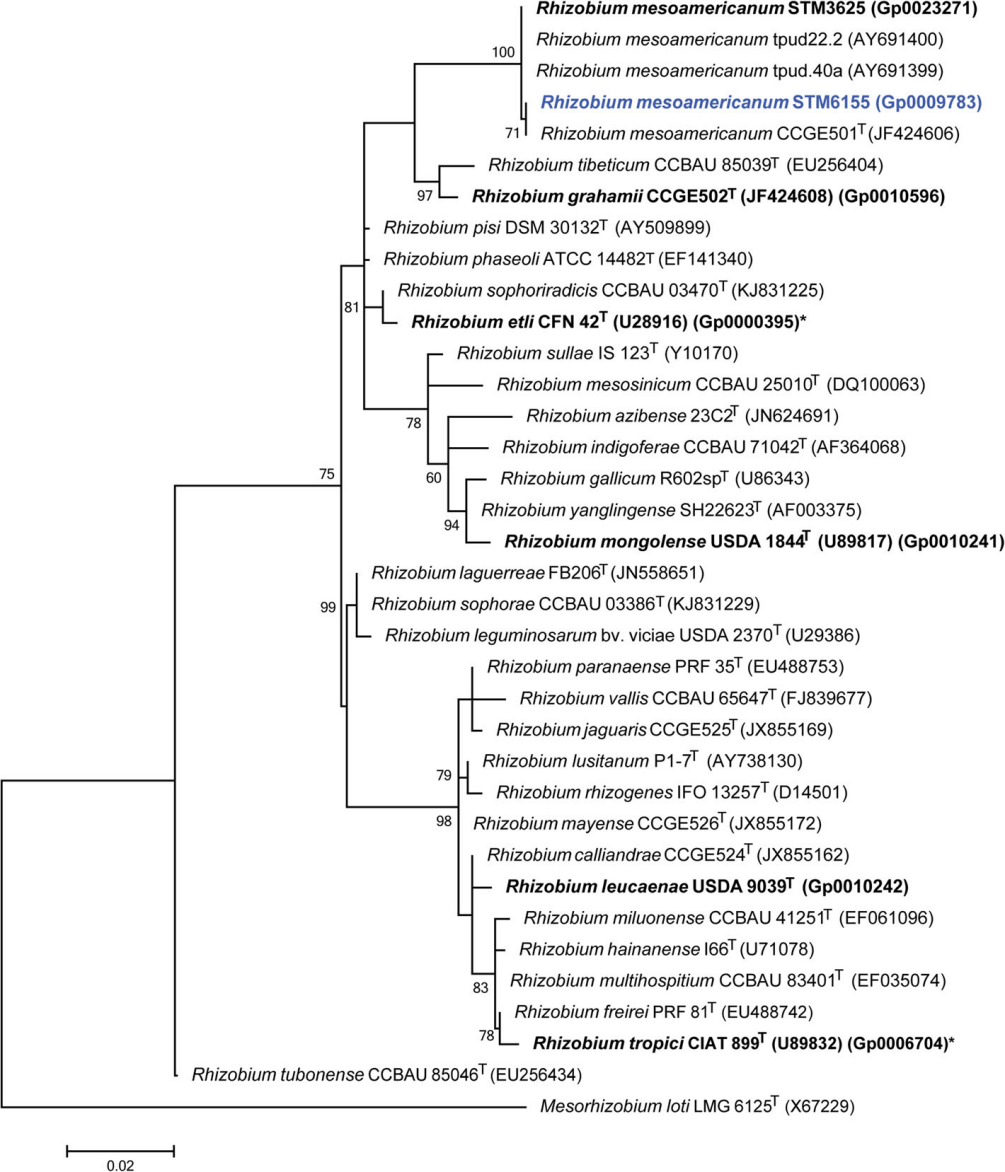
Phylogenetic tree showing the relationship of *Rhizobium mesoamericanum* STM6155 (shown in *bold blue* print) to *Rhizobium* spp. and other root nodule bacteria in the order *Rhizobiales* based on aligned sequences of the 16S rRNA gene (1286 bp intragenic sequence). *Mesorhizobium loti* LMG6125^T^ was used as an outgroup. All sites were informative and there were no gap-containing sites. Phylogenetic analyses were performed using MEGA, version 5 [53]. The tree was built using the Maximum-Likelihood method with the General Time Reversible model [54]. Bootstrap analysis [55] with 500 replicates was performed to assess the support of the clusters. Type strains are indicated with a superscript T. Strains with a genome sequencing project registered in GOLD [31] are in bold font and the GOLD ID is provided after the GenBank accession number, where this is available. Finished genomes are indicated with an asterisk

Figure 2 shows the phylogenetic relationship of *R. mesoamericanum* STM6155 in a 16S rRNA sequence based tree. This strain is the most similar to *R. mesoamericanum* CCGE501^T^ based on the 16S rRNA gene alignment, with sequence identities of 100% over 1362 bp, as determined using the EzTaxon-e database, which contains the sequences of validly published type strains [28]. Minimum Information about the Genome Sequence for STM6155 is provided in Table 1 and Additional file 1: Table S1.

**Table 1:** Classification and general features of *Rhizobium mesoamericanum* STM6155 in accordance with the MIGS recommendations [56] published by the Genome Standards Consortium [57]

MIGS ID	Property	Term	Evidence code^a^
	Classification	Domain Bacteria	TAS [58]
		Phylum *Proteobacteria*	TAS [59, 60]
		Class *Alphaproteobacteria*	TAS [59, 61]
		Order *Rhizobiales*	TAS [62]
		Family *Rhizobiaceae*	TAS [63]
		Genus *Rhizobium*	TAS [15]
		Species *mesoamericanum*	TAS [15, 20]
	Gram stain	Negative	IDA
	Cell shape	Rod	IDA
	Motility	Motile	IDA
	Sporulation	Non-sporulating	NAS
	Temperature range	Mesophile	NAS
	Optimum temperature	28°C	NAS
	pH range; Optimum	7.0	TAS [15, 20]
	Carbon source	Varied; includes mannitol	TAS [15, 20]
MIGS-6	Habitat	Soil, root nodule on host	TAS [15]
MIGS-6.3	Salinity	Up to 1.5% but not 3% NaCl (w/v)	TAS [15, 20]
MIGS-22	Oxygen requirement	Aerobic	TAS [15]
MIGS-15	Biotic relationship	Free-living/symbiont	TAS [15]
MIGS-14	Pathogenicity	Non-pathogenic	NAS
	Biosafety level	1	TAS [64]
	Isolation	Root nodule of *Mimosa pudica* L.	TAS [15]
MIGS-4	Geographic location	Proximity of Mont Dore, New Caledonia	TAS [15]
MIGS-5	Sample collection	2009	TAS [15]
MIGS-4.1	Latitude	166.612297	TAS [15]
MIGS-4.2	Longitude	−22.254586	TAS [15]
MIGS-4.4	Altitude	112 m	TAS [15]

Evidence codes – *IDA* inferred from direct assay, *TAS* traceable author statement (i.e., a direct report exists in the literature), *NAS* non-traceable author statement (i.e., not directly observed for the living, isolated sample, but based on a generally accepted property for the species, or anecdotal evidence)

These evidence codes are from the Gene Ontology project [65, 66]

#### Symbiotaxonomy

*R. mesoamericanum* STM6155 was isolated from nodules of *M. pudica*, as were others members of this species including STM3625, STM3629, tpud40a and tpud22.2 [12, 15, 17]. However, the type strain of the species, CCGE501^T^, originates from nodules of *Phaseolus vulgaris* L. [20]. Strain STM6155 forms nodules and fixes N_2_ with several *Mimosa* species of American origin, including *M. pudica* and *Mimosa* *acustipulata* Benth. It forms white, ineffective nodules on *Mimosa pigra* L. and *Mimosa* *caesalpinifolia* Benth. but is unable to nodulate *Mimosa scabrella* Benth. STM6155 is also able to form nitrogen-fixing nodules on *P. vulgaris* and on a legume, *Acacia spirorbis* Labill., which grows in the same area from which STM6155 originates [15]. The symbiotic characteristics of *R. mesoamericanum* STM6155 on a range of hosts are summarised in Additional file 1: Table S2. *R. mesoamericanum* STM6155 contains a full set of nodulation genes, and exhibits uncommon features, such as the presence of two alleles of the *nodA* gene in its genome, a feature that seems conserved in several strains of the species such as STM3625 [15, 17, 29].

## Genome sequencing information

### Genome project history

This organism was selected for sequencing at the U.S. Department of Energy funded Joint Genome Institute as part of the *Genomic Encyclopedia of Bacteria and Archaea-Root Nodule Bacteria* (GEBA-RNB) project [23, 24]. The root nodule bacteria in this project were selected on the basis of environmental and agricultural relevance to issues in global carbon cycling, alternative energy production, and biogeochemical importance. The genome project is deposited in the Genomes On-Line Database [30] and a high-quality permanent draft genome sequence is deposited in IMG [31]. Sequencing, finishing and annotation were performed by the JGI. A summary of the project information is shown in Table 2.

**Table 2:** Genome sequencing project information for *Rhizobium mesoamericanum* STM6155

MIGS ID	Property	Term
MIGS 31	Finishing quality	High-quality draft
MIGS-28	Libraries used	1x Illumina Std PE library
MIGS 29	Sequencing platforms	Illumina HiSeq 2000
MIGS 31.2	Fold coverage	Illumina: 279x
MIGS 30	Assemblers	Velvet version 1.1.04; Allpaths-LG version r39750
MIGS 32	Gene calling method	Prodigal 1.4
	Locus Tag	YY3 [67]
	Genbank ID	ATYY00000000
	GenBank Date of Release	15^th^ July 2013
	GOLD ID	Gp0009783
	NCBI BIOPROJECT	163057
MIGS 13	Source Material Identifier	STM6155, WSM4584
	Project relevance	Symbiotic N_2_ fixation, agriculture

### Growth conditions and genomic DNA preparation

*Rhizobium mesoamericanum* STM6155 was streaked onto TY solid medium [26] and grown at 28 °C for 3 days to obtain well grown, well separated colonies, then a single colony was selected and used to inoculate 5 ml TY broth medium. The culture was grown for 48 h on a gyratory shaker (200 rpm) at 28 °C. Subsequently 1 ml was used to inoculate 60 ml TY broth medium and the cells were incubated at 28 °C on a gyratory shaker at 200 rpm until an OD_600nm_ of 0.6 was reached. DNA was isolated from 60 ml of cells using a CTAB bacterial genomic DNA isolation method [32]. Final concentration of the DNA was set to 0.5 mg ml^-1^.

### Genome sequencing and assembly

The draft genome of *R. mesoamericanum* STM6155 was generated at the JGI using Illumina technology [33]. An Illumina standard shotgun library was constructed and sequenced using the Illumina HiSeq 2000 platform which generated 14,034,164 reads totaling 2105 Mbp. All general aspects of library construction and sequencing performed at the JGI can be found on the JGI website [34]. All raw Illumina sequence data was passed through DUK, a filtering program developed at JGI, which removes known Illumina sequencing and library preparation artifacts (Mingkun L, Copeland A, Han J. unpublished), providing 12,829,288 trimmed reads totaling 1924 Mbp. The following steps were then performed for assembly: 1) filtered Illumina reads were assembled using Velvet [35] (version 1.1.04); 2) 1–3 Kbp simulated paired end reads were created from Velvet contigs using wgsim [36]; 3) Illumina reads were assembled with simulated read pairs using Allpaths–LG [37] (version r39750). Parameters for assembly steps were: 1) Velvet (velveth: --v --s 51 --e 71 --i 2 --t 1 --f “-shortPaired -fastq $FASTQ” --o “-ins_length 250 -min_contig_lgth 500”); 2) wgsim -e 0 -1 76 -2 76 -r 0 -R 0 -X 0); 3) Allpaths–LG (PrepareAllpathsInputs:PHRED64 = 1 PLOIDY = 1 FRAGCOVERAGE = 125 JUMPCOVERAGE = 25 LONGJUMPCOV = 50, RunAllpath-sLG: THREADS = 8 RUN = stdshredpairs TARGETS = standard VAPIWARNONLY = True OVERWRITE = True). The final draft assembly contained 152 contigs in 147 scaffolds. The total size of the genome is 6.9 Mbp and the final assembly is based on 1924 Mbp of Illumina data, which provides an average 279x coverage of the genome.

### Genome annotation

Genes were identified using Prodigal [38] as part of the DOE-JGI annotation pipeline [39, 40]. The predicted CDSs were translated and used to search the National Center for Biotechnology Information nonredundant database, UniProt, TIGRFam, Pfam, PRIAM, KEGG, COG, and InterPro databases. The tRNAScanSE tool [41] was used to find tRNA genes, whereas ribosomal RNA genes were found by searches against models of the ribosomal RNA genes built from SILVA [42]. Other non–coding RNAs such as the RNA components of the protein secretion complex and the RNase P were identified by searching the genome for the corresponding Rfam profiles using INFERNAL [43]. Additional gene prediction analysis and manual functional annotation was performed within the Integrated Microbial Genomes – Expert Review platform [44] developed by the Joint Genome Institute, Walnut Creek, CA, USA. The annotated genome of *R. mesoamericanum* STM6155 is available in IMG (genome ID = 2513237088).

## Genome properties

**Fig. 3:**
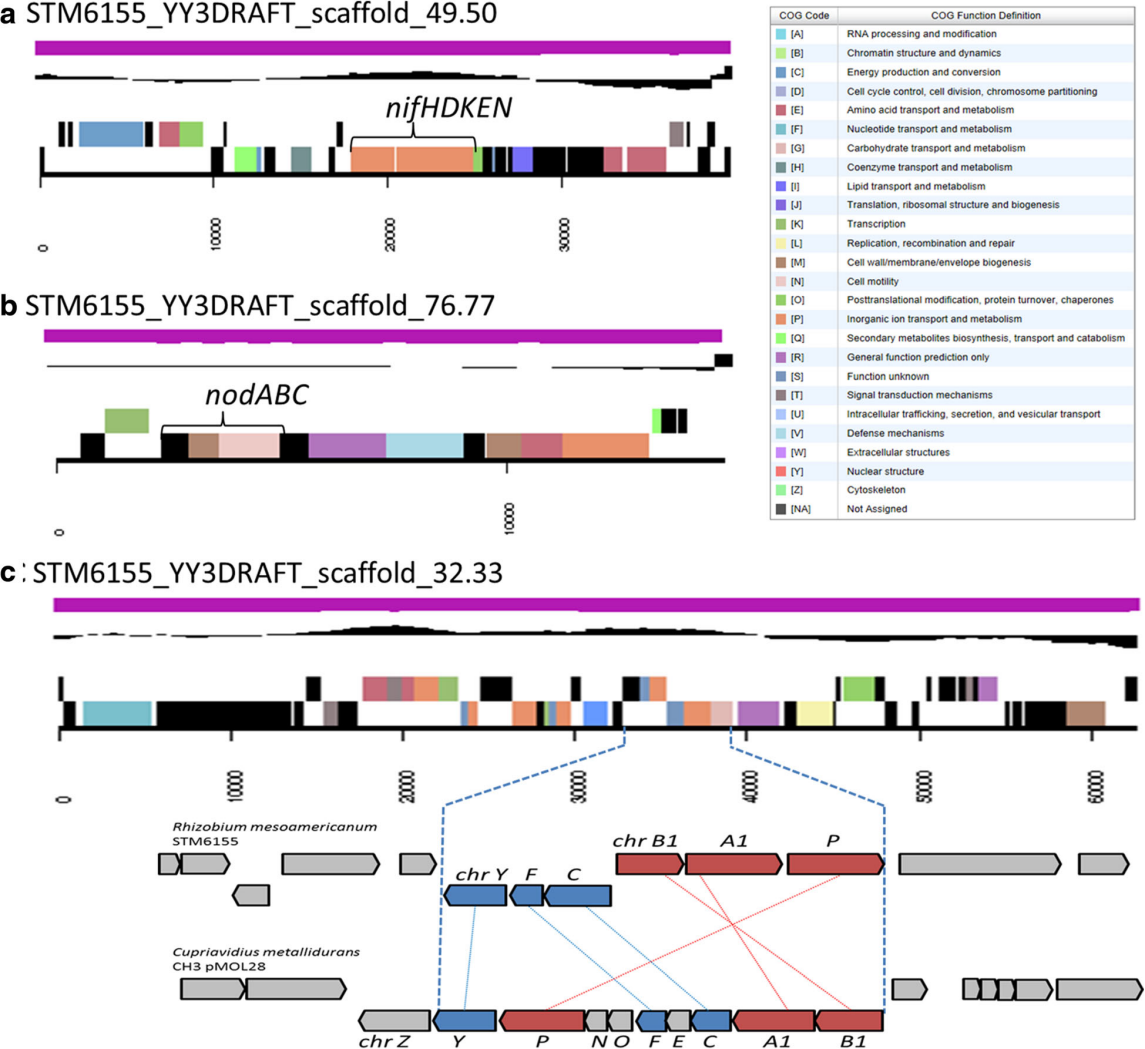
Graphical map of selected scaffolds from the genome of *Rhizobium mesoamericanum* STM6155 containing common nodulation *nodABC* (**a**), nitrogenase *nifHDK* (**b**) and chromate resistance (*chr*) (**c**) clusters. The genes *chrY* to *P* correspond to the STM6155 locus tags YY3DRAFT_04855 to 04860, respectively. From bottom to the top of each scaffold: Genes on forward strand (color by COG categories as denoted by the IMG platform), genes on reverse strand (color by COG categories), RNA genes (tRNAs *green*, sRNAs *red*, other RNAs *black*), GC content, GC skew

The genome is 6,927,906 nucleotides with 58.90% GC content (Table 3) and comprised of 147 scaffolds (selected scaffolds are shown in Fig. 3) of 152 contigs. The location of nodulation (Fig. 3a), nitrogenase (Fig. 3b) and chromate resistance (Fig. 3c) loci on genome scaffolds are shown. From a total of 6926 genes in the genome, 6855 were protein encoding and 71 RNA only encoding genes. The majority of genes (76.02%) were assigned a putative function, whilst the remaining genes were annotated as hypothetical. The distribution of genes into COGs functional categories is presented in Table 4.

**Table 3:** Genome statistics for *Rhizobium mesoamericanum* STM6155

Attribute	Value	% of Total
Genome size (bp)	6,927,906	100.00
DNA coding (bp)	6,004,006	86.66
DNA G + C (bp)	4,080,584	58.90
DNA scaffolds	147	
Total genes	6926	100.00
Protein coding genes	6855	98.97
RNA genes	71	1.03
Pseudo genes	0	0.00
Genes in internal clusters	1382	19.95
Genes with function prediction	5265	76.02
Genes assigned to COGs	4585	66.20
Genes with Pfam domains	5490	79.27
Genes with signal peptides	538	7.77
Genes with transmembrane helices	1529	22.08
CRISPR repeats	0	0.00

**Table 4:** Number of genes of *Rhizobium mesoamericanum* STM6155 associated with general COG functional categories

Code	Value	%age	Description
J	215	4.13	Translation, ribosomal structure and biogenesis
A	0	0.00	RNA processing and modification
K	462	8.87	Transcription
L	141	2.71	Replication, recombination and repair
B	1	0.02	Chromatin structure and dynamics
D	41	0.79	Cell cycle control, cell division, chromosome partitioning
V	120	2.30	Defense mechanisms
T	236	4.53	Signal transduction mechanisms
M	283	5.43	Cell wall/membrane/envelope biogenesis
N	74	1.42	Cell motility
W	17	0.33	Extracellular structures
U	90	1.73	Intracellular trafficking, secretion and vesicular transport
O	191	3.67	Posttranslational modification, protein turnover, chaperones
C	324	6.22	Energy production and conversion
G	463	8.89	Carbohydrate transport and metabolism
E	593	11.38	Amino acid transport and metabolism
F	104	2.00	Nucleotide transport and metabolism
H	248	4.76	Coenzyme transport and metabolism
I	239	4.59	Lipid transport and metabolism
P	259	4.97	Inorganic ion transport and metabolism
Q	162	3.11	Secondary metabolites biosynthesis, transport and catabolism
R	556	10.67	General function prediction only
S	324	6.22	Function unknown
-	2341	33.80	Not in COGs

## Insights from the genome sequence

*R. mesoamericanum* STM6155 shares 100 and 99% sequence identity (over 1346 bp) to the 16S rRNA of the fully sequenced *R. mesoamericanum* type strain CCGE501^T^ [45] and *R. mesoamericanum* strain STM3625 [29], respectively. Moreover the STM6155 genome shows 96.18% average nucleotide identity (ANI) (with 82% of conserved DNA), with the type strain of *R. mesoamericanum* CCGE501^T^ [20], fitting with the species affiliation cut-off defined by Goris et al. (2007) [46] (Table 5).

**Table 5:** Percentage of Average Nucleotide Identities (ANI)^a^ among *Rhizobium* genomes

Strain	CCGE501^T^	STM3625	STM6155	CFN 42^T^	Mim1	CIAT899^T^
*R. mesoamericanum* CCGE501^T^	---	**96.55**	**96.17**	84.28	84.6	84.69
*R. mesoamericanum* STM3625	**96.55**	---	**96.41**	84.4	85.19	85.03
*R. mesoamericanum* STM6155	**96.18**	**96.44**	---	84.45	85.31	84.97
*R. etli* CFN42^T^	84.25	84.4	84.42	---	**98.58**	84.45
*R. etli* bv. *mimosae* Mim1	84.59	85.16	85.3	98.6	---	84.71
*R. tropici* CIAT 899^T^	84.72	85.0	85.03	84.43	84.74	---

^a^ANI values were calculated with jSpecies (based on whole genome Mummer alignments) [68]. Genomes were downloaded from Genbank accessions when already published except *R. mesoamericanum* CCGE501^T^ for which the draft genome was kindly provided by E. Martínez-Romero. Values in bold indicate values above the species cut-off (at least 95% on 69% of conserved DNA) [46]

### Extended insights

**Fig. 4:**
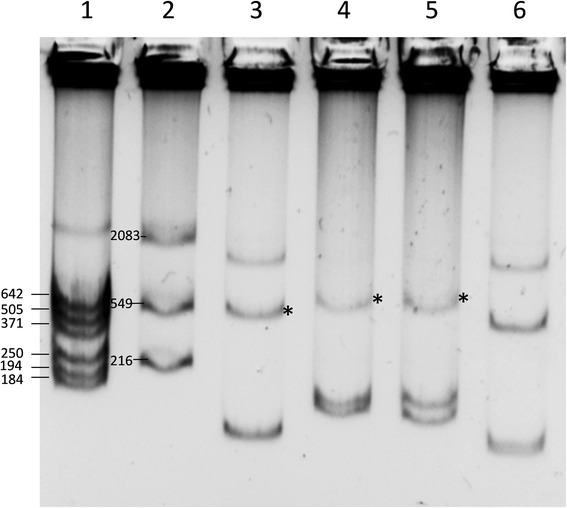
Plasmid profiling of *Rhizobium* strains by the Eckhardt method. Plasmids were run on a 0.9% agarose gel at 5 Volts for 30 min then 60 Volts for 36h in a cold room. Lanes: 1: *R. etli* CFN42^T^ (ladder); 2: *R. tropici* CIAT899^T^ (ladder); 3: *R. mesoamericanum* STM3625 (French Guiana); 4: *R. mesoamericanum* STM3629 (French Guiana), 5: *R. mesoamericanum* STM6155 (New Caledonia); 6: *R. mesoamericanum* CCGE501^T^ (Mexico). The * indicates the symbiotic plasmid

**Fig. 5:**
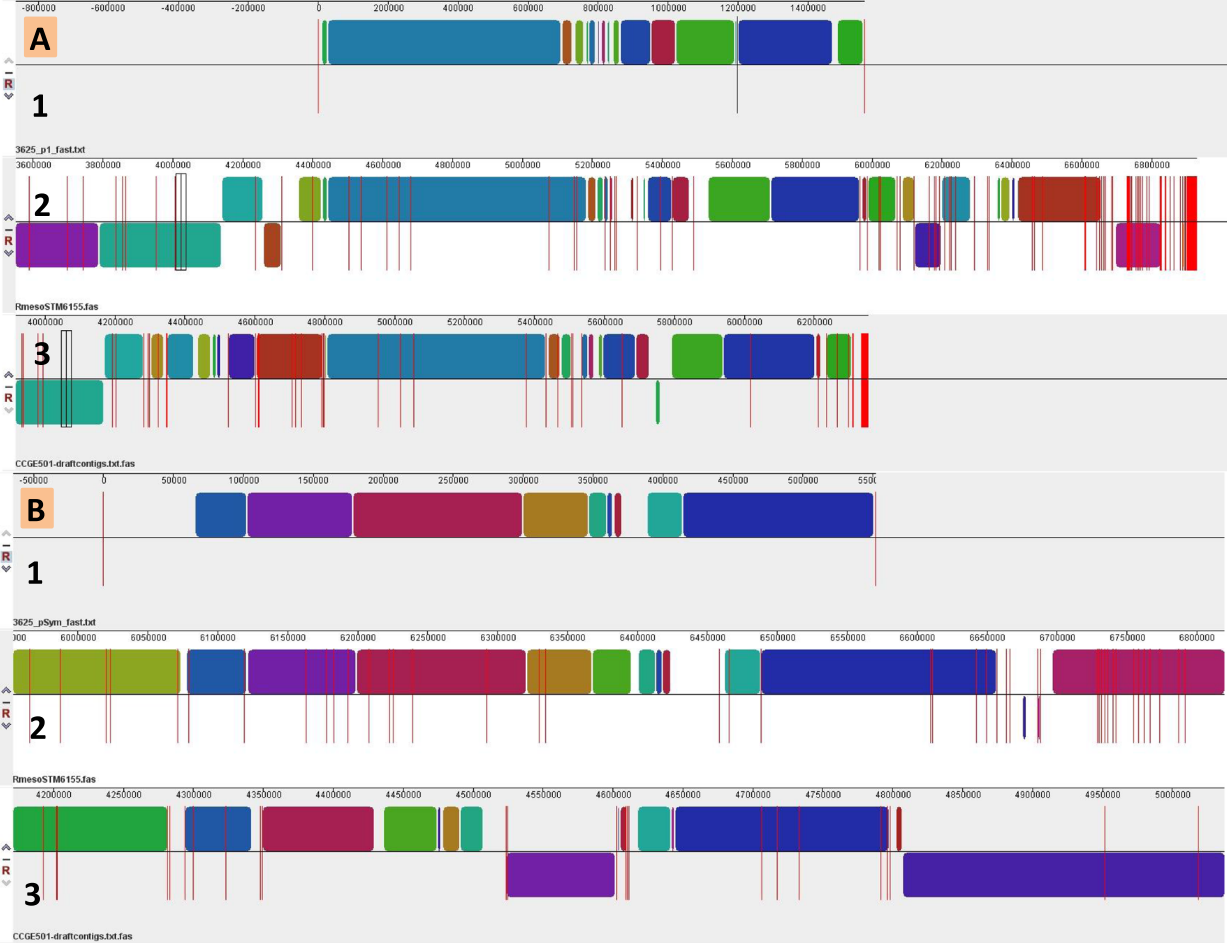
Alignments (using progressive Mauve software) of STM3625 megaplasmid P1 (A1) and pSym (B1) with draft genomes of *R. mesoamericanum* isolates STM6155 (A2, B2) and CCGE501^T^ (A3, B3). The linked blocks in the alignment represent the common local colinear blocks (LCBs) among the compared genomes and homologous blocks among genomes are shown as identically colored regions. The *red lines* in A1 and B1 represent plasmid P1 boundaries (only P1 is shown), while in A2, B2, A3 and B3 they represent contigs boundaries (only homologous contigs to P1/pSym are shown)

We produced plasmid profiles of several *R. mesoamericanum* isolates by the Eckhardt method [47] to compare their plasmid content with genomic data. As shown in Fig. 4, the STM6155 plasmid profile differs from those of STM3625 and CCGE501^T^. Firstly, the STM6155 and STM3629 plasmid profiles suggested the absence of a 1.5 Mbp megaplasmid (P1) observed in CCGE501^T^ and STM3625. The alignment of the megaplasmid P1 sequence of STM3625 with the draft genomes of STM6155 and CCGE501^T^ (Fig. 5a) using progressive Mauve software [48] shows, however, the presence of P1 homologous regions in STM6155 and CCGE501^T^ genomes. This suggests a putative integration of megaplasmid P1 into the bacterial chromosome in STM6155. This phenomenon was already reported in cell siblings of *Ensifer fredii* (formerly *Rhizobium* sp.) NGR234 [49]. The STM6155 plasmid profile suggests thus a diversity of genome architectures at the intra-species level in *R. mesoamericanum*. This diversity is observed among isolates originating from different continents like STM6155 (New Caledonia) and STM3625 (French Guiana), but also among isolates from the same country like STM3625 and STM3629 (both from French Guiana) [15, 17]. Secondly, Fig. 4 shows that STM6155 harbors a ca. 500 Kbp symbiotic plasmid (pSym) of a slightly larger size than those of STM3625 and CCGE501^T^. The alignment of the STM3625 pSym with the draft genomes of STM6155 and CCGE501^T^ (using progressive Mauve, Fig. 5b) confirms the observed pSym size difference, with the presence of additional genomic regions in the STM3625 pSym. Althabegoiti and colleagues [45] have previously observed that there is only 61.4% of conserved DNA (with ANI of 98.07%) between the pSyms of CCGE501^T^ and STM3625. Here we can extend this observation to the STM6155 pSym, which differs from both STM3625 and CCGE501^T^ pSyms.

**Fig. 6:**
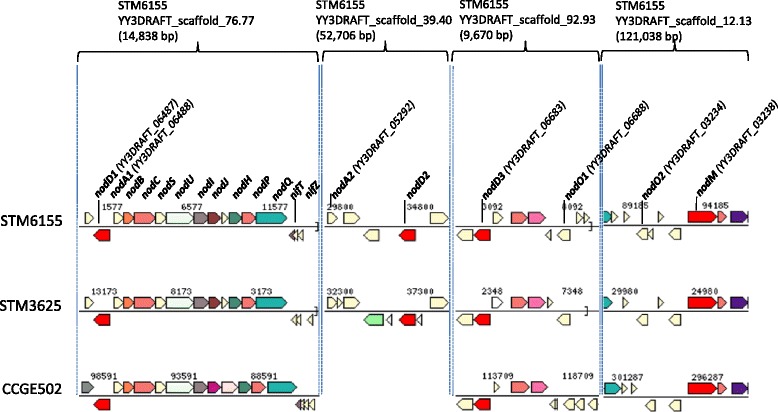
Schematic organization of symbiotic genes conserved in *Rhizobium mesoamericanum* STM3625 and STM6155 and *Rhizobium grahamii* CCGE502^T^

Despite the sequence diversity of the pSyms within *R. mesoamericanum* isolates, the STM6155 symbiosis nodulation genes are highly conserved with those of STM3625 and CCGE501^T^. The STM6155 nodulation genes include *nodA1BCSUIJHPQ*, an additional *nodA* (*nodA2*) gene, three *nodD* (*nodD1, 2* and *3*) transcriptional regulator genes, *nodM*, and 2 *nodO* (*nodO1*, *2*) genes. The gene order is also conserved in *R. grahamii* CCGE502^T^ but this strain does not contain the *nodA2* allele (Fig. 6).

Strain STM6155 was isolated from a nodule of *M. pudica* growing in ultramafic soil at a pH near neutral (pH 6.8) that contained high concentrations of heavy metals, and the highest concentrations of bioavailable chromate among four studied sites [15]. This strain was identified as being resistant to chromate concentrations up to 0.3 mM, that is comparable with chromate tolerance of *Cupriavidus metallidurans* CH34 [15, 50, 51]. Chromate resistance loci (*chr*) have been identified in the heavy-metal-tolerant *C. metallidurans* CH34 and we have discovered orthologs to these genes in STM6155 (Fig. 3c), that were absent from the more chromate sensitive strain *R. mesoamericanum* STM3625. MaGe [52] analysis has revealed synteny of six of the *C. metallidurans* CH34 plasmid-borne *chr* loci in STM6155. However, in contrast to CH3, the loci in STM6155 are arranged into two putative operons, *chrBAP* (locus tags YY3DRAFT_04858 - YY3DRAFT_04860) and *chrCFY* (locus tags YY3DRAFT_04857 - YY3DRAFT_04855) located adjacent to one another on complementary strands.

## Conclusions

*R. mesoamericanum* STM6155 is a microsymbiont of *Mimosa pudica* L. and *Phaseolus vulgaris* L. [9], both of which have centres of origin in central/south America. The genome size of STM6155 is 6.9 Mbp with 58.9% GC content. This strain forms a clique with the two other *R. mesoamericanum* strains STM3625 and CCGE501^T^ based on average nucleotide identity comparisons (species cut-off above 95% on >69% of conserved DNA, as defined by Goris et al. [46]. However, the genome of STM6155 has a different architecture compared with the genomes of STM3625 and CCGE501^T^, with STM6155 lacking a megaplasmid (P1) and containing a different sized pSym and small plasmid. Although STM6155 has a larger pSym, there is a notable symbiotic *nod* gene conservation between the three *R. mesoamericanum* strains, which is also shared with *Rhizobium grahamii* CCGE502^T^ [20]. However, the genomes of the *R. mesoamericanum* strains contain two *nodA* alleles whereas *R. grahamii* CCGE502^T^ genome has only one. Within the STM6155 genome, we have identified a *chr* chromate efflux gene cluster of six genes arranged into two putative operons and we postulate that this cluster is important for the survival of STM6155 in ultramafic soils containing high concentrations of chromate. The availability of sequenced genomes of *R. mesoamericanum* should provide further insights into rhizobial biogeographic distribution and should enable free-living and symbiotic attributes to be compared with those *Mimosa* symbioses induced by beta-rhizobia.

## Additional file

Additional file 1: Table S1. Associated MIGS record for STM6155. **Table S2.** Nodulation and N_2_ fixation properties of *Rhizobium mesoamericanum* STM6155 on selected legume hosts. (DOCX 50 kb)

## Abbreviations

½LA: Half strength lupin agar
ANI: Average nucleotide identity
GEBA-RNB: Genomic encyclopedia for bacteria and Archaea-root nodule bacteria
IMG: Integrated microbial genomes
TY: Tryptone-yeast extract agar
