# Supplementary material for: High-quality draft genome sequence of Rhizobium mesoamericanum strain STM6155, a Mimosa pudica microsymbiont from New Caledonia
Source: Stand Genomic Sci. 2017 Jan 17;12:7. doi: 10.1186/s40793-016-0212-4 (PMC5240323; doi:10.1186/s40793-016-0212-4)
Supplement: Additional file 1: Table S1. — Associated MIGS record for STM6155. Table S2. Nodulation and N2 fixation properties of Rhizobium mesoamericanum STM6155 on selected legume hosts. (DOCX 50 kb) [file 40793_2016_212_MOESM1_ESM.docx]

Additional file 1: **Table S1.** Associated MIGS record for STM6155

| **MIGS-ID** | field name | description |
| --- | --- | --- |
| **MIGS-1** | Submit to INSDC/Trace archives |  |
| **1.1** | PID | Gp0009783 |
| **1.2** | Trace Archive |  |
| **MIGS-2** | MIGS CHECK LIST TYPE |  |
| **MIGS-3** | Project Name | GEBA - Root Nodulating Bacteria |
| **MIGS-4** | Geographic Location | Proximity of Mont Dore, New Caledonia |
| **4.1** | Latitude | -22.254586 |
| **4.2** | Longitude | 166.612297 |
| **4.3** | Depth | 0-10 cm |
| **4.4** | Altitude | 112 m |
| **MIGS-5** | Time of Sample collection | 2009 |
| **MIGS-6** | Habitat (EnvO) | Soil, root nodule, legume host |
| **6.1** | temperature | 28 |
| **6.2** | pH |  |
| **6.3** | salinity |  |
| **6.4** | chlorophyll |  |
| **6.5** | conductivity |  |
|  |  |  |
| **6.6** | light intensity |  |
| **6.7** | dissolved organic carbon (DOC) |  |
| **6.8** | current |  |
| **6.9** | atmospheric data |  |
| **6.10** | density |  |
| **6.11** | alkalinity |  |
| **6.12** | dissolved oxygen |  |
| **6.13** | particulate organic carbon (POC) |  |
| **6.14** | phosphate |  |
| **6.15** | nitrate |  |
| **6.16** | sulfates |  |
| **6.17** | sulfides |  |
| **6.18** | primary production |  |
| **MIGS-7** | Subspecific genetic lineage | *Rhizobium mesoamericanum* STM6155 |
| **MIGS-9** | Number of replicons |  |
| **MIGS-10** | Extrachromosomal elements |  |
| **MIGS-11** | Estimated Size | 6,927,906 bp |
| **MIGS-12** | Reference for biomaterial or Genome report |  |
| **MIGS-13** | Source material identifiers | STM6155 |
| **MIGS-14** | Known Pathogenicity | Non-pathogen |
|  |  |  |
| **MIGS-15** | Biotic Relationship | Symbiotic |
| **MIGS-16** | Specific Host | *Mimosa pudica* |
| **MIGS-17** | Host specificity or range (taxid) | *Miimosa* spp. |
| **MIGS-18** | Health status of Host | Healthy; effective nitrogen fixation |
| **MIGS-19** | Trophic Level |  |
| **MIGS-22** | Relationship to Oxygen | Aerobe |
| **MIGS-23** | Isolation and Growth conditions | TY media, 28°C, aerobic |
| **MIGS-27** | Nucleic acid preparation | CTAB |
| **MIGS-28** | Library construction | Illumina standard PE |
| **28.1** | Library size | 1,924 Mbp |
| **28.2** | Number of trimmed reads | 12,829,288 |
| **28.3** | vector |  |
| **MIGS-29** | Sequencing method | Illumina HiSeq 2000 platform |
| **MIGS-30** | Assembly |  |
| **30.1** | Assembly method | Velvet, version 1.1.04, ALLPATHS r39750 |
| **30.2** | estimated error rate |  |
| **30.3** | method of calculation |  |
| **MIGS-31** | Finishing strategy |  |
| **31.1** | Status | High-quality draft |
| **31.2** | coverage | 279× |
| **31.3** | contigs | 152 |
| **MIGS-32** | Relevant SOPs |  |
| **MIGS-33** | Relevant e-resources |  |

Table S2 Nodulation and N_2_ fixation properties of *Rhizobium mesoamericanum* STM6155 on selected legume hosts

| **Species Name** | **Growth Type** | **Nod** | **Fix** | **Reference** |
| --- | --- | --- | --- | --- |
| *Acacia spirorbis* Labill. (New Caledonia) | Perennial | + | + | [9] |
| *Mimosa acustipulata* Benth. (Brazil) | Perennial | + | + | This study |
| *Mimosa caesalpinifolia* Benth. (Brazil) | Perennial | + (w) | - | This study |
| *Mimosa pigra* L. (French Guiana) | Perennial | + (w) | - | This study |
| *Mimosa pudica* L. commercial | Perennial | + | + | [9] |
| *Mimosa pudica* var. unijuga Duchass. & Walp.) Griseb. (Taiwan) | Perennial | + | + | This study |
| *M. pudica* L. var unknown (French Guiana) | Perennial | + | + | This study |
| *Mimosa scabrella* Benth. (Brazil) | Annual | - | - | This study |
| *Phaseolus vulgaris* L. | Annual | + | + | [9] |

(w) indicates ineffective white nodules
